# Changes of Phenolic Profiles, Bioaccessibility, and Antioxidant Performance of *Areca catechu* L. Extracts During In Vitro Digestion

**DOI:** 10.1002/fsn3.71665

**Published:** 2026-03-22

**Authors:** Wenting Dai, Xiaoning Kang, Jiahui Dai, Shiping Wang, Jianbang Ji

**Affiliations:** ^1^ Institute of Agro‐Products Processing and Design, Hainan Academy of Agricultural Sciences, Haikou Key Laboratory of Areca Processing Research Haikou China; ^2^ Key Laboratory of Tropical Fruit and Vegetable Cold‐Chain of Hainan Province Haikou China; ^3^ Sanya Institute of Hainan Academy of Agricultural Sciences Sanya China

**Keywords:** antioxidant activity, *Areca catechu* L, bioaccessibility, in vitro digestion, phenolic components

## Abstract

Areca nut (Areca *catechu* L.), an economically vital tropical fruit in China, generates substantial agricultural waste with untapped bioactive potential. This study provides new insights into the impacts of in vitro digestion on the phenolic components, bioactivity, and bioaccessibility of extracts from the areca nut fruit (ANF), husk (ANH), and seed (ANS) of areca nut. Remarkably, the ANS extract showed the highest total phenolic content (TPC, 497.99 ± 2.35 mg GAE/g extract DW) and total flavonoid content (TFC, 1341.08 ± 25.15 mg RE/g extract DW), significantly surpassing other samples both before and after in vitro digestion. Areca nut seed extract revealed the strongest antioxidant activity following intestinal digestion. The bioaccessibility of areca polyphenols increased after in vitro digestion, and flavonoids from the extract exhibited significantly higher bioaccessibility (62.23%–73.27%) compared to total phenols. The phenolic profile revealed that flavonoids accounted for the largest proportion, followed by phenolic acids. Importantly, the majority of the identified polyphenols (on average, 26 out of 33 per part) were significantly upregulated during gastrointestinal digestion. The results reveal that areca nuts, particularly the seeds, are a valuable source of bioactive compounds, highlighting their potential as bioactive ingredients for functional food or nutraceutical applications.

## Graphical abstract

This diagram illustrates the dynamic changes in phenolic content, bioaccessibility, antioxidant activity, and phenolic profiles of areca nut extracts from different parts during in vitro digestion, highlighting their functional food potential.

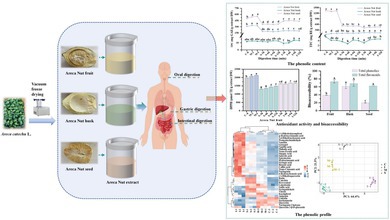

## 1 Introduction

Areca Nut ( *Areca catechu* L.) is a notable tropical fruit primarily cultivated in South and Southeast Asia, with around 700 million people globally consuming it regularly (Guo et al. 2020). More than 95% of areca nut is grown in Hainan Province, China, with an annual production volume reaching approximately 600,000 tons. The areca nut industry plays a pillar role in local economic development (Sun et al. 2023). Areca nut has a long‐standing history of edible and medicinal applications that can kill parasites, treat abdominal pain, bloating, indigestion, and malaria (Yang et al. 2021). Areca nuts are consumed in three main forms: chewing fresh areca nut fruit, dried seeds, and dried areca nut husk. Despite this economic significance, the areca nut industry faces a critical sustainability challenge: processing generates massive quantities of underutilized by‐products, creating both environmental burden and missed opportunities for value creation.

Areca nuts are attributed to various phytochemicals, especially phenolic compounds. The phenolic compounds provide a range of health benefits, including antioxidative, antimicrobial, anti‐inflammatory, mood‐enhancing, and glucose‐regulating effects (Bernardi et al. 2020; Shen et al. 2017; Yang et al. 2021). Two major polyphenols, epicatechin and syringic acid, have been identified in areca seeds through LC‐TOF‐MS analysis, and their potential antioxidant activities have been confirmed (Zhang et al. 2014). By utilizing UHPLC–MS/MS, catechin, procyanidin B1, procyanidin B2, and L‐epicatechin were identified as the key flavonoids in areca nut seed extract, which showed enhanced free radical scavenging effectiveness over flower and husk extracts (Song et al. 2022). The latest research reveals that the anti‐fatigue effect of areca nut polyphenols is attributed to the energy homeostasis and redox balance through the gut‐liver axis (Zhang et al. 2024). These studies emphasize the potential of areca polyphenols as functional components.

Despite these potential benefits of isolated compounds, it is crucial to acknowledge that traditional areca nut consumption is associated with well‐documented health risks, including oral submucous fibrosis and carcinogenicity, particularly when chewed (Ariyawardana et al. 2006; Zhong et al. 2021). Therefore, this study does not advocate areca nut chewing practices. Instead, it explicitly focuses on the characterization and potential value of isolated phenolic extracts derived from processing by‐products, aiming to support the objective evaluation of these bioactive components for potential ingredient development under appropriate safety assessment.

Moreover, the processing and packaging of areca nuts results in the generation of substantial waste materials, including husk, seed, wastewater and fruits that are discarded due to their failure to comply with established commercial quality criteria, imposing substantial costs on areca nut enterprises. Valorizing these by‐products as sources of bioactive ingredients would not only alleviate environmental and economic burdens but also provide an opportunity to develop new foods and ingredients with added nutritional and functional value, in line with “zero waste” and sustainable utilization strategies. In this context, the rich polyphenol content of areca nut makes it a potentially valuable source of natural antioxidants and other bioactive compounds.

Nevertheless, the biological effects of phenolic compounds depend not only on their total content but also on their bioaccessibility following gastrointestinal digestion. Bioaccessibility can be affected by factors such as matrix composition and concentration, release capacity, chemical structure, digestive enzymes, and molecular interactions with other food components, including proteins, lipids, and dietary fibers (de Araújo et al. 2021). Consequently, assessing the stability, transformation, and potential absorption of phenolic compounds within the digestive system is essential for understanding their in vivo relevance (Chait et al. 2020).

Against this background, the specific novelty of this study lies in a systematic comparison of the phenolic profiles and antioxidant properties across different areca nut material streams (including by‐products), coupled with a comprehensive evaluation of their stability and bioaccessibility using an in vitro gastrointestinal digestion model. Unlike previous studies that focused primarily on raw chemical extraction, this work integrates compositional analysis with digestion‐related transformations. The findings aim to provide a theoretical basis for the comprehensive utilization of areca nut as a bioactive resource and for the development of novel food ingredients derived from areca phenolic extracts.

## 2 Materials and Methods

### 2.1 Materials

Areca nuts were from Hainan Nanyin Agricultural Development Co. LTD (Qionghai, China). Pepsin, salivary amylase, pancreatin. Bile salts were supplied by Solarbio Industrial Inc. (Beijing, China). Dialysis bags (MD34‐8000‐14000) were obtained from Yibo Biotechnology Co. Ltd. (Beijing, China). The assay kit for total phenol content and the kit for determining plant flavonoid content, 2, 2′‐dipheny‐1‐picrylhydrazyl, 2, 2′‐azinobis‐(3‐ethylbenzothiazoline‐6‐sulfonic acid), and total antioxidant capacity assay kit with ferric ion‐reducing antioxidant power were procured from Suzhou Michy Biomedical Technology Co. Ltd. (Suzhou, China). All the chemicals and reagents applied in this research conformed to analytical grade specifications.

### 2.2 Sample Preparation

The areca nut was freeze‐dried, and various parts, including areca nut fruit (ANF), areca nut husk (ANH), and areca nut seed (ANS), were individually isolated. The samples were ground into a powder separately, mixed with 80% ethanol solution at a ratio of 1:20 (w/v) per 10 g powder, and sonicated for 20 min. Subsequently, the mixtures were centrifuged at 8000 rpm for 20 min at 4°C. The supernatant was collected, concentrated using a rotary evaporator, freeze‐dried, and stored at −80°C for future use.

### 2.3 In Vitro Digestion Simulation

A standardized three‐step static in vitro digestion model was used to simulate the digestion of areca nut samples in mouth, stomach, and small intestine sequentially, with some modifications, according to Minekus et al. (2014) and Hu et al. (2023). Stock solutions of electrolytes for simulated saliva fluid (SSF), simulated gastric fluid (SGF), and intestinal fluid (SIF) were made by dissolving various salts in deionized water and stirring them continuously overnight.

Initially, 0.6 g of freeze‐dried samples were combined with 30 mL of deionized water and completely dissolved with the aid of ultrasound. After sampling, 6 mL of the sample was blended in a 1:1 ratio with anhydrous ethanol. After centrifugation at 10,000 rpm for 15 min at 4°C, the supernatant was collected and labeled as C‐0. The remaining sample was incubated at 37°C. Approximately 20 mL of the sample solution was added to 20 mL of electrolyte stock solution of SSF, and the pH was adjusted to 7.0 ± 0.1, with 1 M NaOH. Subsequently, α‐amylase and CaCl_2_ were added to the mixture to reach final concentrations of 75 U/mL and 0.75 mM, respectively. Prepared according to the enzymatic activity provided by the supplier. Digestion was carried out immediately at 37°C with agitation at 150 rpm. Samples were collected by oral digestion at 2 and 5 min. Immediately after sampling, an equal volume of anhydrous ethanol was added to terminate the digestive reaction (Ge et al. 2021; Natolino et al. 2024). Finally, the mixtures underwent centrifugation at 10,000 rpm for 15 min at 4°C, and the obtained supernatants were labeled as M‐2 and M‐5, respectively.

After oral digestion, the mixture was supplemented with a 1:1 (v/v) stock solution of SGF, and the pH was adjusted to 3.0 with 3 M HCl. Subsequently, pepsin and CaCl_2_ were introduced, reaching final concentrations of 2000 U/mL and 0.075 mM, respectively. Prepared according to the enzymatic activity provided by the supplier. The mixture was incubated at 37°C for 120 min at 150 rpm during in vitro gastric digestion. Sampling intervals were 30, 60, 90, and 120 min. The samples were thoroughly mixed with an equal volume of anhydrous ethanol to halt the digestion process. Following this, the mixtures were spun at 10,000 rpm for 15 min at 4°C, and the resulting supernatants were separated. The samples were defined as S‐30, S‐60, S‐90, and S‐120, respectively.

The SIF stock solution was combined with the gastric chyme in a 1:1 (v/v) ratio, and the pH was adjusted to 7.0 ± 0.1 using 1 M NaOH. Pancreatin, bile salts, and CaCl_2_ were added to the mixture to achieve final concentrations of 100 U/mL, 10 mM, and 0.3 mM, respectively. The preparation was based on a target trypsin activity of 100 U/mL. For the intestinal digestion phase, the mixture was digested at 37°C for 120 min with agitation at 150 rpm. Sampling occurred at intervals of 30, 60, 90, and 120 min. As mentioned above, the digestion reaction was terminated with anhydrous ethanol, and the supernatants were collected for testing. The samples were named I‐30, I‐60, I‐90, and I‐120, respectively. The above digestions were performed in triplicate with independent sample preparations.

### 2.4 Total Phenolic Content (TPC)

TPC was measured following the method outlined by Xiong et al. (2022) and Sharma et al. (2022). Following this, 10 μL of the sample solution was added to 190 μL of the reaction reagent and left to incubate at room temperature for 10 min. Absorbance was then determined at 765 nm with a microplate reader and the results were expressed as mg gallic acid equivalent (GAE) /g extract DW.

### 2.5 Total Flavonoid Content (TFC)

The TFC was determined following the method described by Naheed et al. (2017). Next, 80 μL of 60% ethanol and 80 μL of the sample solution were mixed with 20 μL of reagent I. After stabilization for 6 min, 20 μL of reagent II was added and allowed to react for 6 min at room temperature. Then, 80 μL of reagent III was mixed with the solution and reacted for 15 min. Absorbance was recorded at 510 nm using a microplate reader. Ethanol (60%) was used as the control. The results were expressed as mg rutin equivalent (RE) /g extract DW.

### 2.6 Assessment of Antioxidant Properties

#### 2.6.1 Determination of Scavenging Ability of DPPH Free Radicals

According to the method reported by Chen et al. (2024), the DPPH scavenging activity was tested. A 20 μL aliquot of 80% ethanol and 20 μL of the sample solution were mixed with 380 μL of DPPH working solution and incubated in darkness for 20 min. Absorbance at 515 nm was measured using a microplate reader, with 80% ethanol as the blank control. The results were expressed as μmol Trolox equivalent (TE)/g extract DW.

#### 2.6.2 Determination of the Scavenging Ability of ABTS Free Radicals

The ABTS free radical scavenging activity was measured using a commercial assay kit (Xiong et al. 2022). Afterward, 10 μL of 80% ethanol and 10 μL of the sample solution were mixed with 190 μL of ABTS working solution and reacted for 20 min. The absorbance was recorded at 734 nm. Ethanol (80%) was used as the control.

#### 2.6.3 FRAP Analysis

The FRAP assay was performed according to the method described by Chen et al. (2024). Next, 10 μL of 80% ethanol and 10 μL of the sample solution were combined with 190 μL of FRAP working solution and incubated at room temperature for 20 min. Absorbance was measured at 593 nm. Ethanol (80%) was used as the control.

### 2.7 Bioaccessibility

The bioaccessibility of areca polyphenols and flavonoids was evaluated using the method described by Hu et al. (2023). The digested final product was added to a dialysis bag with a pore size of 8 kDa, clamped, immersed in Krebs–Ringer solution at 37°C, and stirred at 150 rpm for 3 h. Bioaccessibility was determined using Equation (1).

(1) $$\text{Bioaccessibility}\;\left(\%\right)=\frac{Q_{0}-Q_{t}}{Q_{0}}\times 100\%$$

where *Q*_*0*_ is the initial amount of the target substance added to the dialysis bag (mg), and *Q*_*t*_ is the amount of the remaining sample in the dialysis bag after time *t* (min) (mg).

### 2.8 Identification of Phenolics

Some modifications were made using previous methods (Li et al. 2024). UHPLC (Vanquish, Thermo, United States) and a high‐resolution mass spectrometer (Q Exactive, Thermo, United States) were used to analyze the phenolics in the oral and gastrointestinal digestive products. Phenolics were separated using a Waters HSS T3 column (50 × 2.1 mm, 1.8 μm). The mobile phase comprised 0.1% formic acid in water (A) and 0.1% formic acid in acetonitrile (B), set at a flow rate of 0.3 mL/min. The column temperature was maintained at 40°C, and the injection volume was set to 2 μL. The gradient program was as follows: A/B (90:10, v/v) at 0 min, A/B (90:10, v/v) at 2.0 min, A/B (40:60, v/v) at 6.0 min, A/B (40:60, v/v) at 9.0 min, A/B (90:10, v/v) at 9.1 min, and A/B (90:10, v/v) at 12.0 min. The electrospray ionization source parameters in negative‐ion scan mode were set as follows: ion spray voltage, −2.8 kV; temperature, 350°C; sheath gas pressure, 40 arb; aux gas pressure, 10 arb; capillary temperature, 320°C, and mass scan range, 100–900 m/z.

### 2.9 Statistical Analysis

Post‐digestion, the raw data on polyphenol composition were uploaded to Omicshare (https://omic.sanshugroup.com) for the generation of heatmaps and principal component analysis. The raw data were preprocessed using unit variance standardization. Statistical analysis was performed in OriginPro 2021. One‐way analysis of variance (ANOVA) was used to analyze the data, with statistical significance defined as *p* < 0.05. Pearson's correlation coefficient (*r* ^2^) was employed to assess correlations.

## 3 Results and Discussion

### 3.1 Changes in TPC and TFC

**FIGURE 1:**
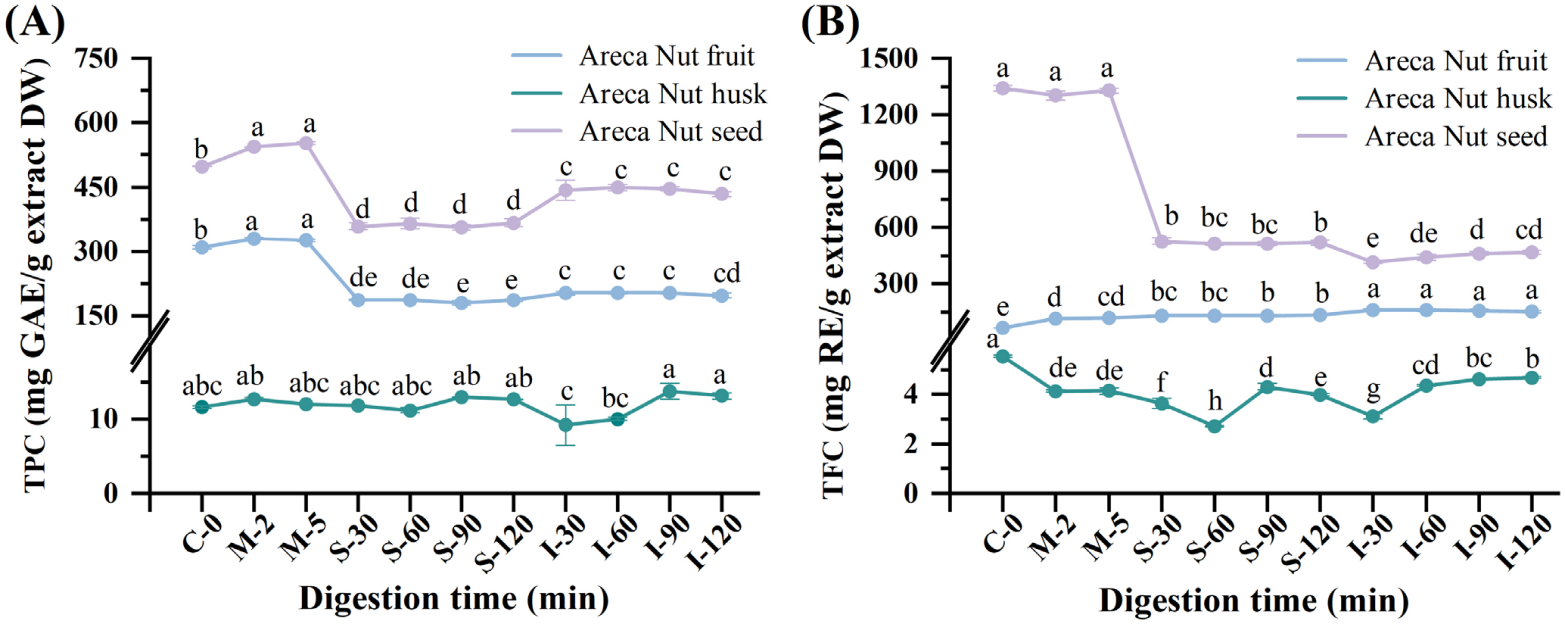
Changes in (A) total phenolic content (TPC) and (B) total flavonoid content (TFC) of ANF, ANH, and ANS during in vitro digestion. Different letters represent significant differences (*p* < 0.05). C‐0: Undigested samples; M‐2: Oral digestion at 2 min; M‐5: Oral digestion at 5 min; S‐30: Gastric digestion at 30 min; S‐60: Gastric digestion at 60 min; S‐90: Gastric digestion at 90 min; S‐120: Gastric digestion at 120 min; I‐30: Intestinal digestion at 30 min; I‐60: Intestinal digestion at 60 min; I‐90: Intestinal digestion at 90 min; I‐120: Intestinal digestion at 120 min.

We quantified the total phenolic content (TPC) and total flavonoid content (TFC) of digested samples from various parts of the areca nut to explore the release patterns of phenolic compounds during in vitro digestion. Figure 1 presents the TPC and TFC of the ANF, ANH, and ANS extracts. The polyphenol content differed significantly among the plant parts (Figure 1A). The TPC for ANF, ANH, and ANS were 309.99 ± 5.96, 11.66 ± 0.33, and 497.99 ± 2.35 mg GAE/g extract DW, respectively. The TPC of ANS was the highest, approximately 42.71‐fold higher than that of ANH, and TPC was approximately 3.6‐fold higher than that of the extracts obtained by Sun et al. (2023). This difference may be due to the source of raw materials, parts, extraction conditions, and different physiological and cytological activities within the plant organs (Cruz‐Carrión et al. 2022; Kim et al. 2024). After short digestion in the mouth, TPC in ANH did not differ significantly (*p* > 0.05); however, TPC in ANF and ANS were released significantly compared to the control (*p* < 0.05). This may be due to mechanical disruption during chewing, which releases some ingredients and increases the surface area for the action of digestive enzymes in the saliva (Velderrain‐Rodríguez et al. 2014). Kaeswurm et al. (2022) discovered that the release of polyphenols in apple peel and flesh during oral digestion was considerable, perhaps due to variations in raw materials and the composition of simulated saliva. Gastrointestinal digestion did not significantly affect the TPC of ANH, primarily ANF and ANS. Following gastric and intestinal digestion, the TPC of ANF decreased by 39.94% and 36.42%, respectively, while the TPC of ANS decreased by 26.33% and 12.83%, respectively, compared to the undigested crude extract. de Paulo Farias et al. (2021) also observed a significant reduction in phenolic content in uvaia seed extract during gastric and intestinal digestion (reduction of 35% and 50%, respectively). Throughout the gastrointestinal digestion process, the released polyphenols may be partially degraded owing to the presence of digestive enzymes (Chen et al. 2024).

Additional reports have demonstrated that phenolics were relatively stable in the gastric digestive phase and decreased significantly in the intestinal digestive phase (Cao et al. 2021; Ma et al. 2023; Wang et al. 2020). In this study, TPC in ANF and ANS increased from the stomach phase to the intestinal phase, indicating that phenolic substances were primarily released during intestinal digestion, similar to the digestion trend of carob polyphenols reported in previous studies (Chait et al. 2020). The notable increase might be attributed to the extended extraction period (an additional 2 h) and the effect of intestinal enzymes on the food structure, promoting the liberation of bound phenolics. On the other hand, differences in phenol release during in vitro simulated digestion are influenced by variations in the food matrix (Xie et al. 2022).

Different tissues of the areca nut also had different levels of flavonoid content. Figure 1B displays that the TFC of ANF, ANH, and ANS were 64.94 ± 0.39, 5.54 ± 0.07, and 1341.08 ± 25.15 mg RE/g extract DW, respectively. The TFC of the ANF extract increased significantly (*p* < 0.05) compared to that of the undigested extract during in vitro simulated digestion, with a 1.81, 2.05, and 2.33‐fold increase observed at the end of the oral, gastric, and intestinal phases, respectively. Similar to the findings with ANF extract, the TFC of dried raspberry fruits and seeds increased by 4.37 and 3.30‐fold, respectively, during in vitro simulated digestion (Qin et al. 2018). The results also displayed that the TFC of ANS extract decreased significantly during in vitro gastrointestinal digestion (−61.31% and −65.04%). The decrease in flavonoid content may have resulted from the partial degradation of some phenolic compounds. The TFC was markedly greater during the gastric digestion stage compared to the intestinal digestion stage. This may be due to the flavonoids having enhanced stability under acidic conditions (Acevedo‐Fani et al. 2021). Although the changes in TFC in the ANH extract fluctuated greatly during digestion, the overall trend was similar to that of the seed extract, and the TFC in the intestinal digestion stage was higher than that in the stomach digestion stage. This may be due to the action of intestinal digestive enzymes and bile salts, which may accelerate the release of flavonoids from the matrix (Wang et al. 2020). Overall, the differences in the released amounts of these active ingredients may be due to differences in the bioactive ingredients and properties of raw materials from different sources (Gullon, Pintado, Fernández‐López, et al. 2015).

### 3.2 Changes in Antioxidant Activity

Antioxidant methods employing various chemical mechanisms may be essential for accurately evaluating the potential of food samples to counteract oxidation (López‐Alarcón and Denicola 2013). In this study, the antioxidant activity of the areca nut extract during in vitro digestion was evaluated using three antioxidant assays: DPPH, ABTS, and FRAP. Because DPPH, ABTS, and FRAP rely on different reaction environments and redox mechanisms, digestion‐induced changes in phenolic speciation may affect these assays differently. DPPH is more sensitive to readily accessible radical‐quenching antioxidants, whereas ABTS is applicable to a broader range of antioxidants and may better reflect compounds released into the aqueous digestive milieu. FRAP reflects overall electron‐donating (reducing) capacity; thus, transformations such as decomplexation, hydrolysis/depolymerization to smaller phenolics, or digestion‐driven changes in phenolic ionization can shift FRAP responses even when total phenolics show similar trends. Therefore, these assays provide complementary insights into the interactions between areca nut extracts and reactive species.

**FIGURE 2:**
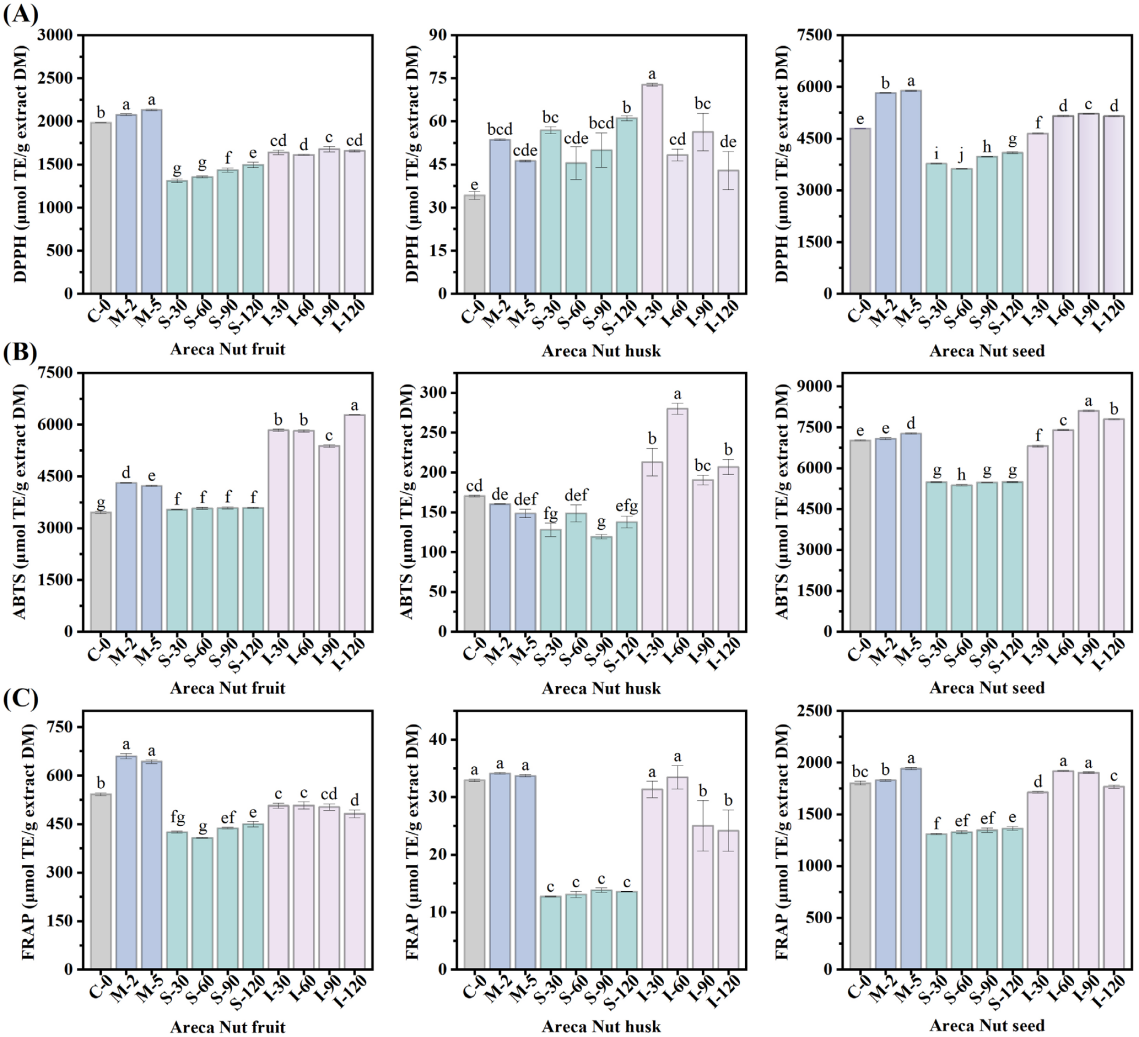
Changes in (A) DPPH radical‐scavenging activity (DPPH), (B) ABTS scavenging activity (ABTS), and (C) Ferric ion reducing antioxidant power (FRAP) in areca nut fruit (ANF), areca nut husk (ANH), and areca nut seed (ANS) extracts during in vitro digestion. Different letters represent significant differences (*p* < 0.05).

The results are presented in Figure 2. The trend of antioxidant activity (DPPH, ABTS, and FRAP) in different parts of the areca nut was similar to that of the TPC (Figure 1A) throughout the simulated in vitro digestion process. The antioxidant activity of the ANH extract was the lowest, while that of the ANS extract was the strongest during in vitro digestion, which may be attributed to the phenolic compound content. The ANH extract exhibited the lowest antioxidant activity throughout in vitro digestion, possibly influenced by its phenolic composition (Xie et al. 2022). Oral digestion positively affected the DPPH, ABTS radical scavenging capacity, and FRAP of the fruit and seed extracts. Compared to the undigested samples, the DPPH, ABTS, and FRAP values of the ANF extract were significantly increased by 7.44%, 22.15%, and 18.52%, respectively (*p* < 0.05), while the corresponding increases in ANS extract were 22.8%, 3.55%, and 7.82%, respectively (*p* < 0.05). These trends were consistent with the changes in TPC (Figure 1A), suggesting that oral digestion contributed to releasing the active components from the areca nut extract (Velderrain‐Rodríguez et al. 2014). Nevertheless, the change of the ANH extract was not significant during oral digestion, consistent with the change in TPC in the corresponding stages.

As shown in Figure 2, simulated gastric digestion reduces the DPPH radical scavenging capacity and FRAP reducing power of ANF and ANS, along with the FRAP reducing power of ANH. By the end of gastric digestion, compared to undigested samples, the DPPH and FRAP values of ANF decreased by 24.65% and 17.11%, respectively, whereas DPPH and FRAP values of ANS decreased by 14.7% and 24.35%, respectively. The FRAP value of ANH decreased by 58.8%, similar to the results for ethanol extracts of passion fruit peel reported by Cao et al. (2021). Mechanistically, the acidic environment and pepsin‐rich conditions may promote phenolic‐protein interactions and precipitation of high‐molecular‐weight tannin‐like compounds, reducing the fraction of freely reactive antioxidants measured by chemical assays. In addition, changes in the structure of antioxidants following digestion may impact their reactivity towards forming nitrogen free radicals, potentially contributing to the decreased DPPH radical scavenging ability (Kamiloglu and Capanoglu 2013). Differences reported across foods (e.g., chestnut skins showing increased DPPH after gastric digestion) further suggest that the gastric effect depends strongly on the phenolic profile and matrix composition (Tu et al. 2021).

Compared to the gastric phase, the DPPH radical scavenging and FRAP reducing abilities of the three extracts exhibited varying degrees of increase in the intestinal phase, similar to the TPC trend observed in this stage. Barreira et al. (2008) indicated that total phenolic content is directly correlated with the antioxidant capacity of plants. The number of phenolic hydroxyl groups increases as monomers or aglycones are released during digestion, and the interaction between phenolic hydroxyl groups as hydrogen donors and free radicals may enhance free radical scavenging capacity. The variation in antioxidant activity, whether a decrease or increase, may be linked to the presence of other substances in the extracts that contribute to this activity, or to the bio‐ or chemical transformation of phenolic compounds, which influences antioxidant activity (Stübler et al. 2019).

The ABTS values of ANF, ANH, and ANS extracts at the end of intestinal digestion were 6285.07 ± 12.24, 206.82 ± 16.58 and 7800.31 ± 35.99 μmol TE/g extract DW, respectively. These values increased by 75.23%, 50.24%, and 41.95%, respectively, compared to those at the end of gastric digestion. Additionally, these values were significantly higher than those of the undigested samples (*p* < 0.05), consistent with the results of a study on apple in vitro digestion by Bouayed et al. (2011). Another study displayed that the ABTS radical scavenging ability of grape polyphenols was significantly improved (*p* < 0.05) under slightly alkaline conditions in the intestinal phase compared to the gastric phase (Tagliazucchi et al. 2010). The shift from an acidic to an alkaline environment may have improved the antioxidant activity of phenolic substances by promoting the deprotonation of hydroxyl groups on the aromatic ring. Alternatively, it could be attributed to structural modifications of polyphenols or the liberation of new compounds with greater antioxidant potential (Bouayed et al. 2011; Djaoudene et al. 2021; Tagliazucchi et al. 2010). These results also revealed a link between the antioxidant activity and phenolic content of extracts from different parts of the areca nut.

The fluctuation in antioxidant capacity during digestion can be attributed to the structural transformation of phenolic compounds induced by pH changes and enzymatic action. In the gastric phase, the acidic environment maintains phenolic compounds in their stable, protonated forms, which may limit their electron‐donating potential in the FRAP assay compared to the intestinal phase. Conversely, the transition to the alkaline intestinal environment promotes the deprotonation of phenolic hydroxyl groups, potentially enhancing their ability to participate in Single Electron Transfer (SET) mechanisms, as reflected in the FRAP results.

It is important to note that while these in vitro assays (DPPH, ABTS, and FRAP) provide valuable insights into the potential chemical antioxidant capacity of areca nut extracts, they do not directly predict in vivo biological efficacy. The actual health benefits in the human body are strictly governed by the bioavailability, metabolic biotransformation (e.g., methylation, glucuronidation), and cellular uptake of these compounds, which cannot be fully replicated by chemical models.

### 3.3 Correlation Between Total Polyphenols Content, Flavonoids Content, and Antioxidant Activity

**FIGURE 3:**
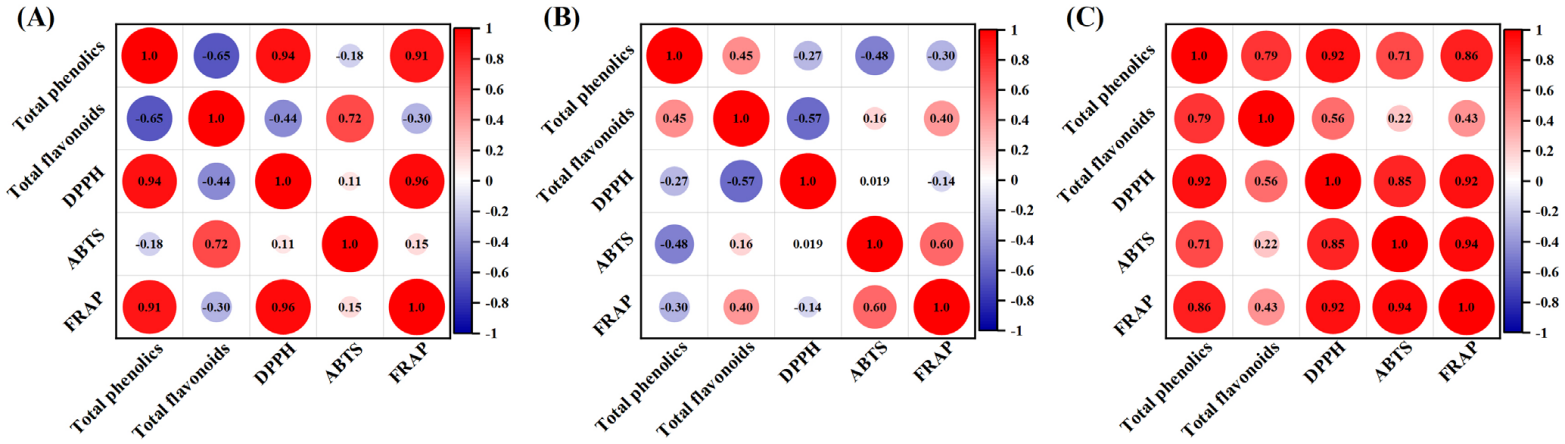
Correlation between total phenolics, total flavonoids, and antioxidant capacity (DPPH, ABTS, and FRAP) in (A) *Areca* nut fruit (ANF), (B) *Areca* nut husk (ANH), and (C) *Areca* nut seed (ANS).

Areca nut is rich in phenols and has good antioxidant activity, and there may be a high correlation between them. Figure 3 illustrates the correlation between phenolic substances and antioxidant capacity in different parts of the areca nut. In ANF extract (Figure 3A), TPC was significantly positively correlated with DPPH radical scavenging ability and FRAP reducing ability (*r* ^2^ > 0.8, *p* < 0.01), and TFC was significantly positively correlated with ABTS radical scavenging ability (*r* ^2^ = 0.70, *p* < 0.05). The findings suggest that the phenolic compounds and flavonoids present in the ANF extracts played a major role in enhancing the antioxidant activity. The TPC of the ANH extract (Figure 3B) was negatively correlated with DPPH, ABTS free radical scavenging ability, and FRAP reducing ability, with *r* ^2^ values of −0.27, −0.48, and −0.30, respectively. Platzer et al. (2022) revealed that a sugar residue at the C‐3 or C‐5 positions of phenolic compounds may reduce antioxidant activity. In contrast, TFC was positively correlated with ABTS radical scavenging and FRAP reducing capacities, suggesting that flavonoids in the ANH extract have ABTS radical scavenging capacity and the ability to reduce Fe^+3^ to Fe^+2^. A high phenol content promotes the free radical scavenging capacity (Ma et al. 2023). The TPC of the ANS extract was significantly positively correlated with the antioxidant capacity (Figure 3C), including DPPH, ABTS, and FRAP (*p* < 0.05), consistent with previous studies (Carbonell‐Capella et al. 2015; Huang et al. 2014). The TFC was slightly positively correlated with the antioxidant capacity of the ANS extract, indicating that phenolic substances and flavonoids in the ANS extract affected the antioxidant activity. However, it should be noted that the observed correlation does not imply exclusive causation. The incomplete correlation implies that, beyond phenolic substances, the complex matrix of the digesta likely contains other non‐phenolic components, such as organic acids, amino acids, small peptides, enzymes, which are also known to influence antioxidant capacity (Chait et al. 2020; Gullon, Pintado, Fernández‐López, et al. 2015). Therefore, while phenolics play a dominant role, the overall antioxidant activity is likely the result of a synergistic effect involving the entire bioactive pool available in the extract.

### 3.4 Bioaccessibility of Polyphenols and Flavonoids Following In Vitro Digestion

**FIGURE 4:**
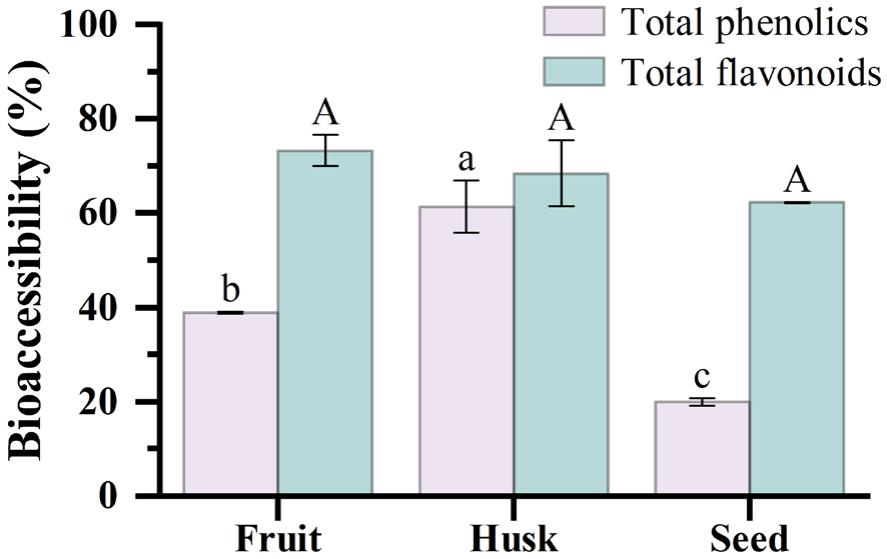
Bioaccessibility of total phenolics and total flavonoids after in vitro digestion of areca nut fruit (ANF), areca nut husk (ANH), and areca nut seed (ANS). Different letters represent significant differences (*p* < 0.05).

The bioavailability of bioactive compounds fundamentally depends on their bioaccessibility. However, it is crucial to note that the dialysis method employed in this study estimates the potential bioaccessibility—the fraction released from the matrix and capable of passing through a semi‐permeable membrane—rather than actual intestinal absorption, which involves complex active transport and metabolic processes. For bioactive compounds to exhibit pharmacological effects, they must be efficiently absorbed from the intestinal tract into the bloodstream and transported to their target sites within the body (de Paulo Farias et al. 2021). At the end of intestinal digestion, the bioaccessibility of TFC in ANF, ANH, and ANS was similar (*p* > 0.05) at 73.27%, 68.43%, and 62.23%, respectively, which was higher than the bioaccessibility of TPC in the corresponding parts (*p* < 0.05). These results were similar to those of a previous study on the bioaccessibility of pomegranate peel flour (Gullon, Pintado, Fernández‐López, et al. 2015). Furthermore, the bioaccessibility of TPC differed significantly among the three groups (*p* < 0.05; Figure 4), with ANH exhibiting the highest bioaccessibility at 61.36%, notably higher by 22.49% and 41.41% than ANF and ANS, respectively. This variation may be attributed to the polyphenol content and composition of the different fractions of areca nut. Phenolic compounds and flavonoids may experience chemical structural changes or engage in various interactions alongside other dietary components throughout the digestive process, potentially influencing their bioaccessibility (Cao et al. 2021), and necessitating further research. Gullon, Pintado, Barber, et al. (2015) also reported that the bioaccessibility of polyphenols in date pit flour and apple bagasse flour reached 78.54% and 91.58%, respectively, by the conclusion of intestinal digestion.

### 3.5 Changes in Major Phenolic Compounds of Areca Nut During In Vitro Digestion

Several bioactive compounds have been identified in areca nut (Song et al. 2022; Zhang et al. 2014). However, the polyphenol composition at specific sites and its changes during in vitro simulated digestion have not been fully analyzed. The content of each polyphenol monomer was measured using UPLC‐MS/MS in negative ion mode to study the polyphenol composition in different parts of the areca nut and the change during in vitro digestion. Table S1 (Supporting Information S1) presents that 34 phenolic metabolites were identified in the ANF extract, 34 in the ANH extract, and 32 in the ANS extract, including the original sample and its digestive products, with flavonoids accounting for the highest proportion, followed by phenolic acids. The primary phenolic compounds of ANF extract were catechin (90.32%), followed by epicatechin (6.48%), similar to the report of Sari et al. (2020). In contrast to the main areca nut extract, the primary phenolic components of ANH extract were catechin (26.75%), luteoloside (26.41%), vanillic acid (9.30%), 4‐hydroxybenzoic acid (9.04%), and vitexin (8.74%). Song et al. (2022) reported that schaftoside and diosmetin were the primary flavonoids of the ANH. Catechin, epicatechin, and rutin were the primary phenolic substances in the ANS extract. Compared to ANF and ANH extracts, the catechin content in ANS extract was the highest, 64.10‐fold higher than that in husk and 1.90‐fold higher than that in fruit. Besides, the predominant phenolic acids identified were vanillic acid and 4‐hydroxybenzoic acid, with their highest concentrations in the ANH extract, similar to the results of Sari et al. (2020).

**FIGURE 5:**
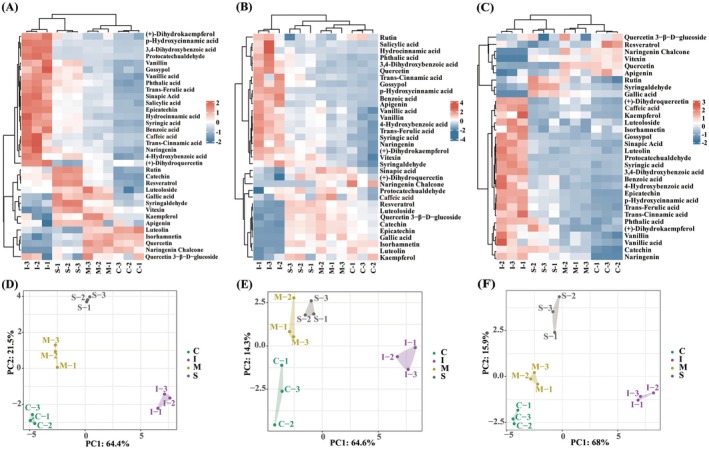
Heatmap of polyphenols in (A) areca nut fruit (ANF), (B) areca nut husk (ANH), and (C) areca nut seed (ANS), and PCA score graphs of polyphenols in (D) areca nut fruit (ANF), (E) areca nut husk (ANH), and (F) areca nut seed (ANS) during in vitro digestion.

A heatmap was generated to visually depict the distinct impact of in vitro digestion on the phenolic profiles of areca nut (Figure 5A–C). The results revealed that most phenolic compounds changed significantly during in vitro simulated digestion and were more abundant than in the original samples, indicating that they were slowly released via the action of digestive enzymes in the simulated digestive system. During gastrointestinal digestion, 27 phenolic metabolites were significantly upregulated in the ANF extract, 24 in the ANH extract, and 26 in the ANS extract. Phenols may interact with the other macronutrients from a matrix or digestive system, such as polysaccharides and digestive enzymes, thereby affecting the stability of these molecules (Chen et al. 2024; Xie et al. 2022). Another possible explanation is that gastrointestinal digestion facilitates the structural transformation and metabolic processes of phenolic compounds (Gao et al. 2022). Previous studies have demonstrated that the upregulation of flavonoids is more prominent after in vitro simulated digestion, similar to the results reported herein (Li et al. 2024; Zhang et al. 2023). Other studies using in vitro digestion and colonic fermentation models have revealed that polyphenols promote health effects by providing absorbable antioxidants and improving the gut microbiota structure (Chen et al. 2024; Xie et al. 2022). This also provides insights for our future research. A few phenolic metabolites, including luteoloside, resveratrol, vitexin, quercetin, and small amounts of luteolin, naringenin chalcone, isorhamnetin, and gallic acid, were significantly downregulated at the end of the intestinal digestion stage, and the degradation of phenolic metabolites in different extracts was different. This may be due to various factors, including different food matrices, chemical reactions such as oxidation and polymerization, pH, and interactions with digestive enzymes and bile salts (de Paulo Farias et al. 2021; Li et al. 2024). PCA revealed a significant separation trend between the three extracts during undigested and in vitro digestion (Figure 5D–F). The PC1 and PC2 contribution rates of the ANF, ANH, and ANS were 85.9%, 78.9%, and 83.9%, respectively. Different regions between the groups were aggregated sufficiently to reflect the differences in phenolic compounds between different digestive stages.

## 4 Conclusions

In summary, extracts from different areca nut fractions showed distinct phenolic profiles, digestion‐driven changes, and antioxidant responses. Among the three fractions, the areca nut seed (ANS) extract consistently had the highest phenolic/flavonoid contents and the strongest antioxidant performance after intestinal digestion, making ANS the most promising fraction for functional ingredient development. Although the husk (ANH) extract showed higher TPC bioaccessibility at the end of digestion, its overall phenolic level and antioxidant capacity were lower than ANS, suggesting that both content and bioaccessibility should be considered when prioritizing raw materials. Practically, areca nut extracts, especially ANS, could be explored as candidate natural antioxidant ingredients for oxidation‐sensitive, lipid‐rich or emulsified products (e.g., edible oils, bakery products, meat/seafood, and beverage emulsions), and areca by‐products may also be evaluated for active packaging/edible coating applications to support by‐product valorization.

In vitro digestion altered both the abundance and composition of phenolics, with many metabolites becoming more detectable during gastrointestinal digestion, suggesting progressive release and conversion along the digestive tract. Flavonoids showed comparatively high estimated bioaccessibility, supporting their potential contribution to post‐digestion antioxidant capacity. However, the present findings are based on chemical antioxidant assays and a dialysis‐based bioaccessibility estimate; therefore, they should be interpreted as in vitro potential rather than direct evidence of in vivo efficacy.

From a practical perspective, these results support the valorization of areca nut processing by‐products, particularly the seed fraction, as a candidate source of phenolic‐rich ingredients for food or nutraceutical formulations, subject to appropriate safety assessment and regulatory compliance. Notably, any potential use as a “natural antioxidant” should be positioned as a possible complement to existing antioxidant strategies in food systems, rather than an unqualified replacement of synthetic additives. Future work should combine digestion with intestinal transport/uptake models (e.g., Caco‐2) and/or in vivo validation, clarify the metabolic fate of key phenolics, and evaluate formulation performance and safety to support application‐oriented development.

## Author Contributions

**Xiaoning Kang:** investigation, methodology, resources. **Wenting Dai:** conceptualization, data curation, writing – original draft, writing – review and editing. **Jianbang Ji:** conceptualization, supervision.

## Funding

This work was supported by The Program of Hainan Association for Science and Technology Plans to Youth R & D Innovation (QCQTXM202202), The Hainan Provincial Natural Science Foundation of China (322QN422), Key Research and Development Project of Hainan Province (ZDYF2024XDNY230), Finance Science and Technology Project of Hainan Province (FW20230002).

## Conflicts of Interest

The authors declare no conflicts of interest.

## Supporting information


**Table S1:** Polyphenols composition of Areca Nut fruit, husk and seed during in vitro digestion.

## Data Availability

The data that support the findings of this study are available on request from the corresponding author. The data are not publicly available due to privacy or ethical restrictions.
