# Supplementary material for: Changes of Phenolic Profiles, Bioaccessibility, and Antioxidant Performance of Areca catechu L. Extracts During In Vitro Digestion
Source: Food Sci Nutr. 2026 Mar 22;14(3):e71665. doi: 10.1002/fsn3.71665 (PMC13093806; doi:10.1002/fsn3.71665)
Supplement: Supplementary file 1 — Table S1: Polyphenols composition of Areca Nut fruit, husk and seed during in vitro digestion. [file FSN3-14-e71665-s001.docx]

**Table S1** Polyphenols composition of Areca Nut fruit, husk and seed during *in vitro* digestion

|  | **Areca Nut fruit extract** | | | | **Areca Nut husk extract** | | | | **Areca Nut seed extract** | | | |
| --- | --- | --- | --- | --- | --- | --- | --- | --- | --- | --- | --- | --- |
| **Phenolic compounds (µg/g)** | C | M | S | I | C | M | S | I | C | M | S | I |
| Gallic acid | 0.08±0.04b | 0.27±0.01a | 0.28±0.05a | 0.04±0.03b | 0.16±0.03a | 0.21±0.05a | 0.18±0.01a | ND | 0.06±0.00c | 0.09±0.00b | 0.12±0.01a | ND |
| 3,4-Dihydroxybenzoic acid | 3.38±0.13c | 4.24±0.08b | 3.85±0.13bc | 10.63±0.47a | 0.91±0.12c | 1.01±0.14c | 1.85±0.24b | 2.92±0.59a | 1.21±0.14b | 1.55±0.24b | 1.56±0.21b | 8.46±0.24a |
| Protocatechualdehyde | 0.61±0.09b | 1.16±0.08b | 0.94±0.11b | 9.8±0.88a | 0.40±0.07a | 0.49±0.04a | 0.47±0.06a | 0.39±0.13a | 0.42±0.03b | 0.75±0.02b | 0.37±0.10b | 9.01±1.33a |
| 4-Hydroxybenzoic acid | 1.19±0.06c | 1.64±0.03b | 1.82±0.1ab | 2.05±0.34a | 37.81±3.60c | 45.53±3.30b | 45.66±1.40b | 56.83±3.71a | 0.48±0.15b | 0.54±0.11b | 1.32±0.38b | 3.34±1.08a |
| Phthalic acid | 23.66±0.59d | 29.42±0.65c | 32.09±0.94b | 37.07±1.62a | 5.88±0.32b | 6.91±0.54b | 9.30±0.97ab | 12.56±3.34a | 1.92±0.26c | 2.07±0.22bc | 2.66±0.57b | 3.46±0.08a |
| Catechin | 3768.08±  60.31d | 5346.30±  89.43c | 6913.34±  105.59a | 5772.77±  146.50b | 111.92±13.34b | 138.48±7.05a | 133.48±3.67a | 37.51±9.67c | 7173.90±  279.18c | 9620.99±  221.83b | 12615.60±  500.12a | 12378.29±  507.43a |
| Vanillic acid | 6.46±0.48d | 9.57±0.18c | 11.53±0.61b | 14.43±0.64a | 38.90±4.02c | 48.37±3.22b | 46.88±1.30b | 55.02±4.48a | 0.14±0.04b | 0.30±0.11ab | 0.44±0.13ab | 0.69±0.43a |
| Caffeic acid | 0.41±0.01b | 0.45±0.05b | 0.5±0.01b | 0.88±0.15a | 0.33±0.05a | 0.39±0.08a | 0.35±0.04a | 0.34±0.06a | 0.26±0.02c | 0.33±0.19b | 0.29±0.02bc | 0.66±0.05a |
| Syringic acid | 2.18±0.08d | 3.26±0.10c | 3.83±0.21b | 5.69±0.45a | 7.75±0.40c | 10.21±0.68b | 10.24±0.89b | 13.16±0.72a | 0.32±0.07b | 0.42±0.02b | 0.44±0.04b | 1.31±0.10a |
| Epicatechin | 270.24±1.85d | 334.39±4.48c | 393.18±3.28b | 641.55±3.51a | 23.37±2.17c | 30.41±1.46a | 27.76±1.00b | 1.95±0.38d | 385.97±1.2d | 486.71±9.14c | 589.56±26.14b | 847.49±4.01a |
| Vanillin | 0.98± 0.04c | 1.30±0.06c | 1.88±0.24b | 2.43±0.31a | 6.57±0.84c | 7.88±0.92bc | 8.37±0.20b | 10.64±0.96a | 0.25±0.06c | 0.41±0.08bc | 0.53±0.03ab | 0.70±0.18a |
| p-Hydroxycinnamic Acid | 1.54±0.02c | 2.19±0.12b | 2.37±0.22b | 7.1±0.14a | 2.69±0.34c | 3.52±0.21b | 3.64±0.24b | 12.34±0.57a | 1.58±0.03d | 2.03±0.09c | 2.44±0.07b | 3.90±0.10a |
| Syringaldehyde | 4.72±0.18c | 5.90±0.25b | 8.43±0.24a | 4.54±0.50c | 2.12±0.22c | 2.63±0.09b | 2.96±0.07ab | 3.08±0.37a | 5.83±0.31bc | 6.47±0.29b | 9.43±0.25a | 5.39±0.49c |
| Rutin | 28.16±0.3c | 30.58±0.66b | 33.61±0.15a | 30.51±0.8b | 2.05±0.11ab | 1.90±0.10ab | 1.85±0.17b | 2.11±0.11a | 55.89±1.66c | 62.06±3.17ab | 66.59±2.64a | 60.64±2.39bc |
| Vitexin | 9.30±0.22c | 10.39±0.33b | 11.91±0.42a | 6.24±0.48d | 36.59±4.23b | 43.45±3.55a | 48.24±2.17a | 49.65±3.09a | 0.45±0.03a | 0.36±0.05b | 0.39±0.02ab | ND |
| Salicylic acid | 0.53±0.07c | 0.85±0.04b | 0.92±0.01b | 1.62±0.08a | 0.57±0.12b | 0.94±0.33b | 0.94±0.24b | 3.57±2.05a | ND | ND | ND | ND |
| Trans-Ferulic acid | 1.96±0.02d | 2.72±0.03c | 3.29±0.07b | 4.38±0.25a | 7.57±1.00c | 9.21±0.43b | 9.49±0.22b | 12.23±1.10a | 1.26±0.09c | 1.62±0.15c | 2.23±0.18b | 3.23±0.37a |
| Sinapic Acid | 0.98± 0.00c | 1.36±0.07b | 1.58±0.05b | 2.48±0.30a | 4.37±0.60bc | 5.52±0.24a | 4.85±0.31ab | 3.95±0.45c | 0.78±0.08c | 1.13±0.04bc | 1.39±0.15b | 3.25±0.37a |
| Quercetin 3-β-D-glucoside | 1.25±0.04a | 1.27±0.04a | 1.21±0.04a | 1.23±0.08a | 0.59±0.12b | 0.78±0.09a | 0.74±0.06a | 0.20±0.06c | 0.63±0.02a | 0.62±0.06a | 0.60±0.05a | 0.61±0.02a |
| Luteoloside | 15.77±0.44c | 17.84±0.61a | 17.53±0.07ab | 16.98±0.08b | 110.53±6.08c | 128.63±8.95b | 139.46±1.36a | 59.13±1.09d | 0.09±0.08b | 0.72±0.16b | 0.81±0.21b | 1.46±0.15a |
| (+)-Dihydroquercetin | 2.33±0.06b | 2.30±0.07b | 1.97±0.05c | 2.90±0.07a | 0.06±0.02a | 0.07±0.00a | 0.07±0.01a | 0.02±0.02b | 0.66±0.02c | 0.86±0.05b | 0.70±0.05c | 1.14±0.01a |
| Benzoic acid | 2.46±0.27c | 5.32±0.40c | 10.83±1.51b | 21.11±3.57a | 2.46±0.16c | 3.19±0.33bc | 4.89±0.57b | 11.80±2.04a | 0.46±0.16c | 1.36±0.42bc | 2.85±1.12b | 6.32±1.74a |
| (+)-Dihydrokaempferol | 0.30±0.02c | 0.34±0.04bc | 0.37±0.03b | 0.47±0.02a | 0.10±0.04c | 0.11±0.01c | 0.16±0.02b | 0.20±0.01a | 0.26±0.03b | 0.24±0.02b | 0.45±0.08a | 0.43±0.06a |
| Resveratrol | 21.70±0.44d | 25.09±0.30c | 30.45±0.18a | 26.10±0.28b | 10.79±0.52c | 12.7±0.82b | 14.09±0.44a | 6.75±0.61d | 0.56±0.05a | 0.47±0.01b | 0.44±0.02b | 0.48±0.04b |
| Luteolin | 0.62±0.02a | 0.55±0.00b | 0.40±0.04c | 0.45±0.03c | 1.30±0.13a | 1.27±0.11a | 1.18±0.02a | 0.66±0.06b | 0.08±0.01b | 0.09±0.00b | 0.09±0.01b | 0.15±0.01a |
| Quercetin | 0.52±0.04b | 0.71±0.04a | 0.20±0.02c | 0.02±0.01d | 0.18±0.01c | 0.19±0.02bc | 0.31±0.06b | 0.55±0.12a | 1.12±0.06b | 1.21±0.05a | 0.61±0.02c | 0.61±0.01c |
| Hydrocinnamic acid | 0.06±0.01c | 0.11±0.04c | 0.34±0.02b | 0.74±0.16a | 0.08±0.01b | 0.09±0.02b | 0.14±0.11b | 0.43±0.36a | ND | ND | ND | ND |
| Trans-Cinnamic acid | 0.30±0.01b | 0.37±0.03b | 0.39±0.03b | 0.71±0.18a | 0.31±0.04b | 0.43±0.03b | 0.56±0.17ab | 0.87±0.37a | 0.37±0.02c | 0.43±0.01bc | 0.54±0.06b | 0.84±0.14a |
| Naringenin Chalcone | 0.21±0.04a | 0.24±0.02a | 0.15±0.02b | ND | 0.06±0.03a | 0.03±0.01b | ND | ND | 0.15±0.01a | 0.15±0.04a | 0.08±0.02b | ND |
| Naringenin | 0.13±0.01c | 0.17±0.01b | 0.19±0.02b | 0.26±0.04a | 0.11±0.01c | 0.14±0.02bc | 0.15±0.01ab | 0.19±0.03a | 0.09±0.00c | 0.16±0.01b | 0.17±0.01b | 0.21± 0.01a |
| Apigenin | 0.64±0.02a | 0.65±0.04a | 0.67±0.00a | 0.62±0.04a | 0.50±0.02b | 0.55±0.06b | 0.54±0.02b | 0.77±0.06a | 0.03±0.00a | 0.03±0.01ab | 0.02±0.00bc | 0.01±0.01c |
| Kaempferol | 0.05±0.00b | 0.08±0.01a | 0.06±0.00ab | 0.03±0.03b | 0.06±0.01a | 0.05±0.01a | 0.05±0.02a | 0.03±0.02a | 0.08±0.00b | 0.14±0.01b | 0.09±0.00b | 0.19±0.06a |
| Isorhamnetin | 0.07±0.00a | 0.07±0.01a | 0.04±0.00b | 0.02±0.01c | 0.03±0.00a | 0.03±0.00a | 0.03±0.01a | ND | 0.06±0.01b | 0.04±0.01b | 0.05±0.01b | 0.09±0.02a |
| Gossypol | 1.06±0.22c | 1.36±0.21c | 2.30±0.10b | 3.32±0.75a | 1.32±0.31c | 1.21±0.29c | 5.43±0.28b | 9.14±2.20a | 2.57±0.63b | 1.76±0.23b | 2.68±0.64b | 11.34±1.71a |

Different letters indicate that the same phenolic compounds from the same areca nut extract differ significantly at different stages of digestion (*p* < 0.05); ND: Not detected. Each value is expressed as mean ± standard deviation (n = 3).
